# On what motivates us: a detailed review of intrinsic *v.* extrinsic motivation

**DOI:** 10.1017/S0033291722001611

**Published:** 2022-07

**Authors:** Laurel S. Morris, Mora M. Grehl, Sarah B. Rutter, Marishka Mehta, Margaret L. Westwater

**Affiliations:** 1 Department of Psychiatry, Depression and Anxiety Center for Discovery and Treatment, Icahn School of Medicine at Mount Sinai, New York, NY 10029 USA; 2 Department of Psychology, Temple University, Philadelphia, PA 19122 USA; 3 Department of Radiology and Biomedical Imaging, Yale School of Medicine, New Haven, CT 06510 USA

**Keywords:** Cognitive models, Depression, Extrinsic motivation, Intrinsic motivation

## Abstract

Motivational processes underlie behaviors that enrich the human experience, and impairments in motivation are commonly observed in psychiatric illness. While motivated behavior is often examined with respect to extrinsic reinforcers, not all actions are driven by reactions to external stimuli; some are driven by ‘intrinsic’ motivation. Intrinsically motivated behaviors are computationally similar to extrinsically motivated behaviors, in that they strive to maximize reward value and minimize punishment. However, our understanding of the neurocognitive mechanisms that underlie intrinsically motivated behavior remains limited. Dysfunction in intrinsic motivation represents an important trans-diagnostic facet of psychiatric symptomology, but due to a lack of clear consensus, the contribution of intrinsic motivation to psychopathology remains poorly understood. This review aims to provide an overview of the conceptualization, measurement, and neurobiology of intrinsic motivation, providing a framework for understanding its potential contributions to psychopathology and its treatment. Distinctions between intrinsic and extrinsic motivation are discussed, including divergence in the types of associated rewards or outcomes that drive behavioral action and choice. A useful framework for understanding intrinsic motivation, and thus separating it from extrinsic motivation, is developed and suggestions for optimization of paradigms to measure intrinsic motivation are proposed.

## Introduction

Motivation is an integral component of human experience. Children spontaneously explore novel items, and adults autonomously engage in new hobbies, even in the absence of clear extrinsic reinforcers. Thus, not all actions are driven by tangible external stimuli or outcomes, known as ‘extrinsic’ motivation, but are driven by more internal drivers, known as ‘intrinsic’ motivation, where the activity is perceived as its own outcome.

Intrinsically motivated behaviors are computationally similar to extrinsically motivated behaviors, in that they strive to maximize goal attainment and minimize punishment, represented mathematically as value and effort cost functions, respectively (Gottlieb, Lopes, & Oudeyer, 2016). However, subjective internal value functions are difficult to characterize, and our understanding of how they are computed and integrated is limited (Gottlieb et al., 2016).

Dysfunction in intrinsic motivation represents an important transdiagnostic facet of psychiatric symptomology, which is often classified as distinct psychological constructs, such as apathy in neurological disorders, anhedonia in depression, and negative symptoms in schizophrenia. Each of these symptom domains may be underpinned by a shared dysfunction of intrinsic motivation, and interventions targeting intrinsic motivation have the potential to improve treatment outcomes for affected individuals.

However, due to a lack of clear consensus, the contribution of intrinsic motivation to psychiatric disorders remains poorly understood. This review aims to provide an overview of the conceptualization, measurement, and neurobiology of intrinsic motivation, providing a framework for understanding the potential contributions to psychopathology and its treatment.

## Historical conceptualizations of intrinsic motivation

During the early 20th century, prominent descriptions of motivation were at odds with each other. Woodworth (1918) suggested that intrinsic motivation governed activities perpetuated by their own ‘native drive’, whereas Thorndike (1911) and Watson (1913) argued that external stimuli governed behavior. Also centered on internal drives, Hull's (1943) ‘drive theory’ posited that all behaviors were performed to seek or avoid primary biological states, including hunger or pain. However, the drive theory could not explain many behavioral anomalies, such as hungry rats withstanding painful electric shocks to explore a novel environment (Nissen, 1930), or rhesus monkeys performing a puzzle task for no biological reason or external reinforcer (Harlow, 1950). By narrowly presuming that biological states drive all behavior, drive theory failed to account for instances in which an organism prioritizes higher-order cognitive drives over physiological ones.

The shortcomings of drive theory led to the emergence of alternate theories of intrinsic motivation. Some argued that homeostatic maintenance of optimal biological or cognitive states (Hebb, 1955; McClelland & Clark, 1953; McClelland, Atkinson, Clark, & Lowell, 1967), or mitigation of incongruency or uncertainty (Festinger, 1957; Kagan, 1972), drove behavior. However, these theories emphasized external stimuli or cognitive representations of external goal states as key drivers of behavior. In the mid-to-late 20th century, several models underscored the importance of novelty-seeking, interest, and autonomy in driving intrinsic motivation. Novelty-seeking was suggested to energize approach behavior via curiosity and exploration that leads to skill mastery, information attainment, or learning (Kaplan & Oudeyer, 2007). Interest and enjoyment in an activity might boost intrinsic motivation by engendering ‘flow’, a prolonged state of focus and enjoyment during task engagement that stretches one's skillset (Csikszentmihalyi, 1975; Nakamura & Csikszentmihalyi, 2009). Finally, self-determination theory (Deci & Ryan, 1980) proposed that human needs for competence, achievement, and autonomy drive intrinsic motivation, aligning with observations that intrinsic motivation stems from an internal perceived autonomy during task engagement (DeCharms, 1968; Lamal, 2003). These models highlight the role of achievement and perceived autonomy (DeCharms, 1968) in driving intrinsic motivation, coinciding with current computational frameworks of intrinsic reward (Chew, Blain, Dolan, & Rutledge, 2021; Murayama, Matsumoto, Izuma, & Matsumoto, 2010).

## The introduction of external goals: a shift to extrinsic motivation

While intrinsic motivation has been proposed to be divorced from external reinforcers, our understanding of motivation has been led largely by using external reinforcers as conceptual and experimental tools. Here, we briefly review historical perspectives on external drivers of motivated behavior, outlining prominent goal- and action-focused models of extrinsic motivation.

Early psychological models of extrinsic motivation suggested that ‘will’ and ‘intention’ fostered goal achievement, emphasizing the influence of goal expectation on action and control (Lewin, 1951; Tolman, 1932). Within this framework, environmental features, as well as an individual's internal state or memory, determine their actions when pursuing a goal, or, more specifically, the cognitive representation of a goal (Kagan, 1972). This requires multiple cognitive representations to be developed, maintained, and updated, with a particular reliance on external stimuli and learning (Deci, Koestner, & Ryan, 1999; Kagan, 1972; Kagan & Moss, 1983).

Alongside psychological model development, economic models of motivation emerged. These models propose that extrinsic goals, or incentives, elicit motivated behavior via a cost-benefit analysis, where motivated choice occurs when benefits outweigh costs. More recently, behavioral economics has considered how individual personality traits, biases, and irrationalities influence motivated behavior (Strombach, Strang, Park, & Kenning, 2016). A recent model (Strombach et al., 2016) incorporates various factors into the classical cost-benefit analysis, including traditional intrinsic (e.g. satisfaction) and extrinsic drivers (e.g. money), with negative influences from costs (e.g. effort, pain), which are merged into a single dynamic, subjective and state-dependent factor that drives motivated behavior. Though this approach is powerful, the explicit focus on incentives provides limited explanatory power for various paradoxical behaviors, including rodents overcoming the high cost to self-stimulate certain brain regions (e.g. nucleus accumbens; Nac) or extrinsic reinforcers' dampening effect on intrinsic motivation.

In reinforcement learning models of decision-making, an organism, or agent, learns which actions maximize total reward. This process has been formalized within computational sciences and modern artificial intelligence systems (Sutton & Barto, 1981; Witten, 1977), where learning and decision-making depend on an extrinsic outcome. One theory suggests that motivated action is driven solely by a need to reduce reward prediction errors (RPEs; Kaplan and Oudeyer, 2007), or the mismatch between expectation and outcome (Montague, Dayan, & Sejnowski, 1996; Schultz et al., 1997). RPEs can also be conceptualized as valuation signals for novel outcomes or unexpected stimuli. RPE-based learning then drives motivated behavior, or action choice, but even if the agent displays intact encoding of action or outcome value, motivated behavior can be dampened by reduced novelty. This highlights the role of novelty, expectation and prediction in learning *per se*, rather than choice valuation.

In action-focused models of motivation, incentives can trigger approach or avoidance behavior by signaling a potential goal state (Berridge, Robinson, & Aldridge, 2009). Incentive motivation thus relies on expectancy, probability, and value of outcomes, which are thought to dictate behavioral choice and decision-making. While greater reliance on stimulus-outcome rather than stimulus-response contingencies has led some to describe incentive motivation as proactive (Beckmann & Heckhausen, 2018), others have characterized it as reactive due to the central role of learning from past experience (Bolles, 1972). Reliance on an expected outcome was central to behaviorism (Watson, 1913, 1930) and operant conditioning (Skinner, 1938), which assume that actions are driven by a reinforcer, and instrumental value is assigned to the behavior itself. Stimulus-response pairs dominate behaviorism and modern theories of habitual behavior (Gläscher, Daw, Dayan, & O'Doherty, 2010), where the dependency on previously reinforced actions ultimately governs motivated choice (de Wit et al., 2011; Gillan, Robbins, Sahakian, van den Heuvel, & van Wingen, 2016; Voon et al., 2014). However, this renders behaviors as repetitive, insensitive to punishment and divorced from goals (Robbins, Gillan, Smith, de Wit, & Ersche, 2012). Therefore, these action-focused models of motivated behavior almost entirely discount intrinsic motivation since extrinsic motivators usurp control of behavior.

Several limitations of extrinsic motivation models must be considered when attempting to characterize intrinsic motivation. First, for cost-benefit analysis and reinforcement learning, an internal representation of the outcome must first be learned, which requires previous experience of the goal. However, intrinsic motivation can occur for novel outcomes, or behaviors that are uncertain or ambiguous. Second, motivation can occur for activities that may already be fully predictable, marking a significant limitation for reinforcement-learning models of motivation, which assume that reward prediction errors drive learning for motivated action. Third, these frameworks cannot fully explain spontaneous novelty seeking or exploratory behavior, in which no external reward is expected and no cost is overcome (Deci et al., 1999; Marsden, Ma, Deci, Ryan, & Chiu, 2014).

## Separating and integrating intrinsic and extrinsic motivation

A key question is whether intrinsic and extrinsic motivation can, or should, be experimentally or theoretically separated. There is some evidence that they are dissociable constructs at the neural level. The most compelling support comes from case reports of patients with basal ganglia lesions who developed ‘psychic akinesia’, a syndrome characterized by difficulty with self-generated action initiation but no difficulty in performing complex cognitive or motor tasks when prompted (Laplane, Baulac, Widlocher, & Dubois, 1984; Lugaresi, Montagna, Morreale, & Gallassi, 1990). In patients with alien hand syndrome, medial prefrontal and anterior cingulate cortex (ACC) lesions lead to a loss of intentional motor control, whereas (pre)-supplementary motor area lesions lead to impairments in implementing motor intentions (Brugger, Galovic, Weder, & Kägi, 2015; Nachev, Kennard, & Husain, 2008). Preclinical findings further show that photostimulation of GABAergic amygdala projections modulates extrinsic motivation without affecting intrinsically motivated behavior (Seo et al., 2016). Together, these findings suggest that intrinsic and extrinsic motivation reflect different cortico-striatal-limbic circuits.

Behavioral research primarily supports the view that intrinsic and extrinsic motivation are partially distinct, interacting processes. For example, if the motivation for intrinsic and extrinsic goals were independent constructs, they might demonstrate an additive or subtractive effect on each other (Woodworth, 1921). Indeed, the expectation (Liu & Hou, 2017) and experience (Badami, VaezMousavi, Wulf, & Namazizadeh, 2011) of an extrinsic reinforcer can increase intrinsic motivation. However, reports of the ‘undermining effect’, in which an external reinforcer reduces intrinsic motivation (Cerasoli, Nicklin, & Ford, 2014; Deci, 1971; Deci, Benware, & Landy, 1974; Lepper & Greene, 1978; Lepper, Greene, & Nisbett, 1973) have sparked debate over how extrinsic reinforcers affect internally-motived behaviors (Cameron & Pierce, 2002; Lamal, 2003; Lepper, Keavney, & Drake, 1996). One explanation for the undermining effect suggests that the presence of an external reinforcer shifts one's perception of the locus of control over the behavior from internal to external (Deci & Ryan, 1980). This implicates a key role of agency, or the belief of action ownership, in intrinsic motivation. While controversial, mounting evidence supports this account of the undermining effect, where various extrinsic motivators (e.g. food, social observation; Ryan, 1982) decrease intrinsic motivation when their delivery is contingent on task-performance.

**Fig. 1.**
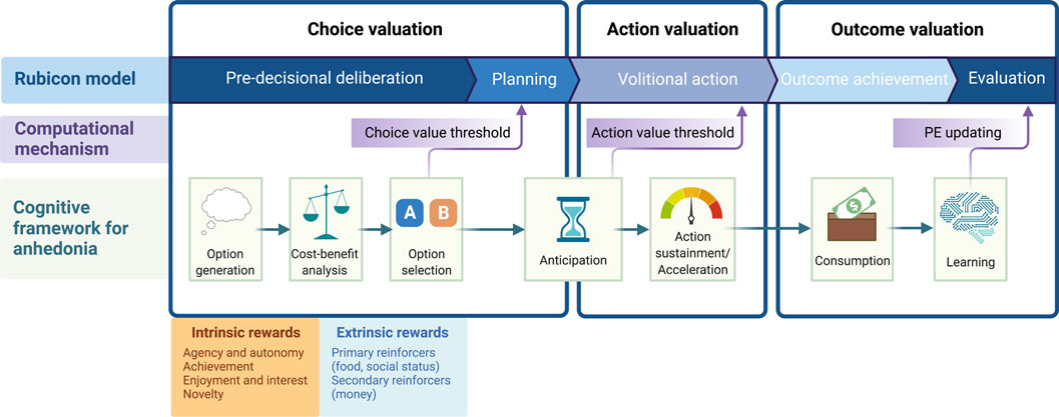
*Schematic framework for parsing motivated action.* Motivated decision-making and action is parsed into separate phases of choice, action and outcome valuation, combining and building upon separate frameworks including the Rubicon model of action phases, well-established computational mechanisms and a recent cognitive framework describing anhedonia and apathy. During choice valuation, pre-decisional deliberation includes option generation, a cost-benefit analysis and option selection. Intrinsic and extrinsic motivation diverges during this early choice valuation phase. Once choice valuation has been computed and an option selected, planning and anticipation occurs. During action valuation, volitional action is initiated and action sustainment or acceleration is maintained. During outcome valuation, outcome achievement and consumption ensue, followed by evaluation based on learning via prediction error (PE) updating. Created with BioRender.com.

A useful framework for parsing motivated action into intrinsic and extrinsic is the Rubicon model of action phases (Heckhausen & Heckhausen, 2018; Heckhausen, 1989). Within this framework, pre-decisional option deliberation occurs, which is followed by choice intention formation and planning, volitional action, outcome achievement, and evaluation (Fig. 1). Husain and Roiser (2018) recently proposed a complementary model to deconstruct apathy and anhedonia into underlying cognitive processes, including option generation, anticipation, action initiation, prediction, consumption and learning. This parcellation broadly reflects the five main stages of the Rubicon model: (1) pre-decisional deliberation (*option generation*); (2) intention formation, planning, initiation (*anticipation*); (3) volitional action (*action initiation, prediction*), (4) outcome achievement (*consumption*); and (5) evaluation (*learning*; Figure 1). Within these overlapping frameworks, the initial pre-decisional deliberation/option generation phase represents the point at which intrinsic and extrinsic facets of motivation diverge, as early drivers of behavior can be intrinsic (e.g. enjoyment, interest, exploration) or extrinsic (e.g. social reward). The differences between these early drivers highlight a key distinction between intrinsic and extrinsic motivation, in which the former is a fundamentally proactive process and the latter reactive.

If a behavior were intrinsically motivated, the pre-decisional deliberation phase might be determined by biological drives, the need to restore homeostasis (Hebb, 1949; Hull, 1943), or a state of incongruency resolution (Festinger, 1957; Kagan, 1972) as described by early theories of intrinsic motivation. In contemporary frameworks, novelty-seeking, exploration, or interest in learning or achievement would render subsequent actions as intrinsically motivated. If a behavior were extrinsically motivated, this pre-decisional deliberation phase would represent the cost-benefit analysis in economic models, prediction-error minimization in reinforcement learning, or effort-reward trade-off computation. Under incentive motivation and behaviorist theories, the pre-decisional deliberation phase would be triggered by conditioned stimuli making conscious deliberation unnecessary and inefficient.

A combination of intrinsic and extrinsic factors likely enters into the pre-decisional deliberation phase to guide motivated behavior (Fig. 1). Although intrinsic and extrinsic motivation are conceptually distinct processes, attempts to formally define them have identified several mechanisms by which they interact, leading to questions about their dissociability. Since they can interact in an additive or subtractive fashion, they may indeed be separate, independent drivers of behavior that are amalgamated during a pre-decisional deliberation phase of behavioral choice.

## Measuring intrinsic motivation

### Human behavior

Early attempts to quantify intrinsic motivation were largely based on behavioral observation, wherein intrinsic motivation was measured as free choice of an activity in the absence of an external stimulus or performance rating (Butler & Nisan, 1986; Daniel & Esser, 1980; Liu & Hou, 2017). These studies also implemented self-report measures of participants' interest or enjoyment in an activity. While such measures do capture intrinsic motivation as inherent task enjoyment, they are limited by their qualitative and indirect nature, as well as by variability in participant insight. However, more objective measures are difficult to develop due to the inherently unobservable nature of intrinsic motivation.

Since spontaneous novelty-seeking and exploratory behavior reflect intrinsic motivation, one candidate objective measure may be the explore-exploit paradigm (Gittins & Jones, 1979; Robbins, 1952). In explore-exploit foraging tasks, participants must choose among various options and either exploit a previously reinforced choice or explore a novel alternative option. An individual's tendency to either explore an environment or exploit their pre-existing knowledge is influenced by perseverance (Von Culin et al., 2014), which acts as an indicator of confidence in the absence of immediate reward. Healthy adults flexibly employ a mix of exploitative and exploratory choices, where striatal and prefrontal dopamine signaling is proposed to drive exploration and exploitation, respectively (Badre, Doll, Long,, & Frank,, 2012; Daw, O'Doherty, Dayan, Seymour, & Dolan, 2006; Mansouri, Koechlin, Rosa, & Buckley, 2017). While these tasks capture one's willingness to trade-off exploratory *v.* exploitative behaviors, they do not measure free-choice exploratory behavior in the absence of explicit reinforcers, which would be most consistent with intrinsic motivation.

Paradigms that allow an individual to choose to explore an environment without extrinsic reinforcers, or to engage in a previously enjoyable or interesting activity, would more closely index intrinsic motivation. Additionally, outcomes that relate to achievement or autonomy, without socially rewarding feedback or monetary outcomes, would also putatively engage intrinsic motivation. Task parameters related to exploration, enjoyment, achievement, and autonomy can each be modulated and computationally modeled to determine their effects on free choice or behavioral activation vigor.

Current computational approaches depend on modeling decision-making, outcome learning, or action-outcome associations to drive our understanding of motivation. Traditional decision-making models often rely on softmax functions to compute values of available actions (Wilson & Collins, 2019), where action selection is based on the ‘policy’ of the best outcome. Computationally, an action selection process computes the probability of an action occurring in any state and the expected reward. A policy is developed based on the assumption that motivated actions are performed to increase the probability of rewards and decrease the probability of punishment. Yet, in everyday life, our actions can be motivated by an arbitrary cue that may signal an internal rewarding state. For example, a standard algorithm solving for motivated action assumes that all actions have equal probability, yet this discounts the unknown drivers and evaluators of internal rewards. Hence, they act as limiting factors to the applicability of decision-making models in studies of intrinsic motivation.

### Neuroimaging

Functional neuroimaging [e.g., functional magnetic resonance imaging (fMRI), electroencephalography (EEG)] offers a measurement modality that may be particularly apt for the study of internally driven processes like intrinsic motivation. Research using fMRI has characterized the neural correlates of various internal processes that lack clear behavioral indicators (e.g. rumination, emotion regulation, pain perception; Zhou et al., 2020; Wagner, N'Diaye, Ethofer, and Vuilleumier, 2011), yet few studies have assessed the neural correlates of intrinsic motivation in humans, which likely reflects the limitations in its behavioral measurement. Studies have largely assessed intrinsic motivation via comparisons with neural responses to extrinsic reinforcers during fMRI, which can be correlated with self-reported intrinsic motivation (Bengtsson, Lau, & Passingham, 2009; Chew et al., 2021; Linke et al., 2010). Despite the relative paucity of neuroimaging studies that clearly separate intrinsic *v.* extrinsic motivation, existing work provides preliminary insight into the neural circuitry of intrinsic motivation.

First, extrinsic reinforcers have elicited amygdala, ACC, ventromedial prefrontal cortex (vmPFC), orbitofrontal cortex (OFC), and ventral striatal (VS) or Nac activity in healthy subjects that was associated with higher self-reported extrinsic motivation but lower self-reported intrinsic motivation (Linke et al., 2010). This could suggest that intrinsic motivation relates to a lower sensitivity of these regions to extrinsic reinforcers, general deactivation of these regions, or that the dampening impact of extrinsic reinforcers on intrinsic motivation is subserved by these regions. Others report that intrinsic motivation (operationalized as the amount of free-time spent on a puzzle-task, which did not relate to task enjoyment, interest, or accuracy), was associated with deactivation in the amygdala, dorsal ACC, dorsomedial striatum, and insula during puzzle-task onset (Marsden et al., 2014). This is another piece of evidence linking neural *deactivation* to intrinsic motivation; however, since these tasks were not related to traditional ‘intrinsic motivators’ like task enjoyment, findings may relate to boredom-reduction behavior that might be more related to punishment avoidance rather than intrinsic motivation *per se*.

Bengtsson et al. (2009) operationalized intrinsic motivation as task-performance with and without explicit experimental observation during fMRI scanning, which boosted self-reported intrinsic motivation. The authors found greater neural activation of ACC, OFC, and lateral prefrontal cortex during task-performance errors when participants were observed (Bengtsson et al., 2009). While implicating a similar network of brain regions as prior studies, these findings cannot be divorced from error-related neural activation modulated by task salience (e.g. observed *v.* not).

In contrast, Murayama *et al*. (2010) provide a more optimal operationalization of intrinsic motivation, in which participants performed a task that was previously rated as inherently interesting, and successful task performance served as the intrinsic reward. During fMRI scanning, feedback for both extrinsic (monetary feedback) and intrinsic (accuracy feedback) rewards elicited VS activation. Participants then had the option to perform the same task without feedback, and intrinsic motivation was operationalized as time spent on the second version of the task. During the second session, VS activation was only diminished for extrinsic rewards, which could reflect reduced VS habituation to intrinsic rewards (Murayama et al., 2010, 2015). Additionally, greater reductions in neural responses to extrinsic reinforcers were related to lower intrinsic motivation (i.e. task engagement time outside of the scanner), suggesting that neural habituation to extrinsic reinforcers may relate to lower intrinsic motivation. A recent computational neuroimaging study modeled intrinsic rewards as successful spatial-motor task performance without experienced errors, which was divorced from learning (Chew et al., 2021). This modeling of intrinsic rewards was akin to the accuracy feedback operationalization of Murayama *et al*. (2010). Both extrinsic (monetary) reward and intrinsic performance-based rewards (successful task completion) recruited vmPFC activation, which related to subjective happiness (Chew et al., 2021). Although limited in their ability to dissociate activation from task performance *per se* and explicit feedback related to achievement, these studies are the closest examples of objective measures of intrinsic motivation, and they suggest that putative reward-processing regions (VS, vmPFC) encode intrinsic rewards.

Complementary studies have examined how curiosity, or the intrinsic motivation to learn, modulates neural responses and influences memory recall (Gruber, Gelman, & Ranganath, 2014; Kang et al., 2009). High-curiosity states augment midbrain and *v.* activity (Gruber et al., 2014), as well as bilateral caudate (Kang et al., 2009) and anterior insula (Lee & Reeve, 2017) responses, which may improve learning and memory. As these paradigms index intrinsic motivation independently from a rewarding outcome, they perhaps provide the strongest support for partially overlapping circuits of extrinsic and intrinsic motivation.

### Dopamine

The brain's dopamine system supports a range of appetitive and aversive motivational processes, including behavioral activation, exertion of effort, and sustained task engagement (Diederen & Fletcher, 2020; Salamone, Yohn, López-Cruz, San Miguel, & Correa, 2016). The mesolimbic pathway, projecting from the ventral tegmental area (VTA) to limbic regions, including the Nac, amygdala, and hippocampus, facilitates reinforcement and associative learning by acting as a ‘Go’ signal for foraging or exploration (Huang, Lv, & Wu, 2016). Although it has long been known that dopamine transmission subserves motivational processes, some evidence suggests that it is particularly important for intrinsic motivation. For example, mesolimbic dopamine contributes to exploration for the sake of interest (DeYoung, 2013; Panksepp & Moskal, 2008), and novel and unexpected stimuli elicit phasic dopamine spikes in rodents (Fiorillo, 2003; Hooks & Kalivas, 1994; Schultz, 1998). In patients with depression, deep-brain stimulation of dopaminergic brain regions including the Nac (Schlaepfer et al., 2007) and the mesolimbic dopamine projections from the VTA (Fenoy et al., 2018) increased subjective interest in, and motivational energy for, previously enjoyable activities (Schlaepfer et al., 2007). Dopamine has also been associated with intrinsically motivated flow states (de Manzano et al., 2013; Gyurkovics et al., 2016).

However, since VTA dopamine spiking is reduced for expected events (Schultz, 1998), it may not be a strong candidate neural mechanism for intrinsic motivation, which can occur for predictable activities. Efforts to reconcile the role of dopamine in learning and motivation suggest that while phasic cell *firing* signals RPEs (Kim et al., 2020), phasic dopamine *release* and local modulation in key regions, such as the VS/NAc, relates to approach motivation (Berke, 2018; Mohebi et al., 2019). Indeed, while VTA dopamine cell firing occurs during reward prediction, only NAc dopamine release covaries with reward availability and ramps up during approach and consumption of reward (Mohebi et al., 2019). Moreover, increasing dopamine in rodents increases their willingness to exert effort, and this has since been replicated across species, including via pharmacological manipulation in humans (Salamone, Correa, Farrar, & Mingote, 2007; Treadway & Zald, 2011). This suggests that, while VTA dopamine spiking underpins reward prediction and learning, it is local NAc dopamine release that encodes motivational drive.

### Opioids, norepinephrine, and related neurotransmitter systems

Though a comprehensive account of the neurotransmitter systems subserving motivated behavior is beyond the scope of this review, we note that endogenous opioid and cannabinoid systems may uniquely modulate intrinsically motivated behavior. For example, mu- and delta-opioid receptor activation underlies the pleasurable effects of opioid and non-opioid drugs of abuse (Berrendero, Robledo, Trigo, Martín-García, & Maldonado, 2010; Trigo, Martin-García, Berrendero, Robledo, & Maldonado, 2010), as well as primary reinforcers (Hsu et al., 2013; Kelley & Berridge, 2002). Activation of mu-opioid receptors has also been shown to mediate motivational states following delta-9-tetrahydrocannabinol (THC) administration in rodents (Ghozland et al., 2002), likely via interactions with the mesolimbic dopamine system. Further evidence implicates antidepressant effects of endogenous opioids in both animals and humans (Peciña et al., 2018), which many partly reflect improved intrinsic motivation (e.g. time mice spent swimming during the forced swim test; Kastin, Scollan, Ehrensing, Schally, and Coy, 1978). Additionally, the endocannabinoid system interacts with both endogenous opioid and dopaminergic systems to influence intrinsic motivation, such as social play (Trezza et al., 2012; Trezza & Vanderschuren, 2008), and voluntary exercise, in rodents (Dubreucq, Koehl, Abrous, Marsicano, & Chaouloff, 2010). Since these systems have been primarily examined in animal models, pharmacological manipulation in humans would be an important next step in delineating the contribution of opioid and endocannabinoid systems to intrinsic *v.* extrinsic motivation.

#### Intrinsic motivation and psychiatry: focus on anhedonia

Problems with motivation are observed across many neuropsychiatric disorders, and these often correspond to distinct symptoms (Table 1). This section focuses on anhedonia, a reduced ability to experience pleasure (Ribot, 1986), as a prevalent clinical manifestation of deficient intrinsic and extrinsic motivation.

**Table 1.** Explicit studies of ‘intrinsic motivation’ in neuropsychiatric disorders

Disorder	Related symptom	Cohort	Measure	Evidence	Reference
Depressive disorders	Anhedonia	*N* = 537 undergraduate students	Motivated Strategies for Learning Questionnaire, 9-item intrinsic value subscale, Pintrich and De Groot (1990).	Academic IM was negatively associated with depression and stress.	Huang et al. (2016)
		*N* = 95 MDD	Autonomous and Controlled Motivations for Treatment Questionnaire.	Autonomous motivation predicted a higher probability of remission and lower post-treatment depression severity among patients across three outpatient treatments: 16 sessions of manualized interpersonal therapy, cognitive–behavior therapy, or pharmacotherapy with clinical management.	Zuroff et al. (2007)
		*N* = 59 subthreshold MDD	Performance of a stopwatch task based on intrinsic motivation during fMRI scanning	Behavioral activation therapy (identify and complete enjoyable activities that provide a sense of achievement) increased activation and connectivity in frontostriatal regions, associated with improved sensitivity to rewards.	Mori et al. (2018)
		*N* = 106 healthy volunteers	Intrinsic Motivation Inventory: two items from the interest/enjoyment subscale.	Participants who were unable to differentiate between positive emotions had stronger links between positive emotions and intrinsic motivation, whereas subjects that were able to differentiate between negative emotions showed a weaker link between negative emotions and intrinsic motivation.	Vandercammen, Hofmans, and Theuns (2014)
		*N* = 33 treatment resistant MDD	Intrinsic Motivation Inventory.	Examined the effectiveness of cognitive remediation with supplemental Internet-based homework, Treatment consisted of 10 weeks of weekly group sessions and daily online cognitive exercises completed at home. Homework completion was associated with worse depressive symptoms and not intrinsic motivation.	Bowie et al. (2013)
		*N* = 300 working adults	Rated 10 job aspects on 6-point scales related in intrinsic (e.g. self growth) and extrinsic (e.g. pay, social status) job features.	Intrinsic work motivation was associated with higher job satisfaction. Higher extrinsic motivation was associated with higher depression scores.	Lu (1999)
		*N* = 215 elite team-sport athletes	Sport Motivation Scale II, Perceived Motivational Climate in Sport Questionnaire II, Basic Need Satisfaction in Sport Scale.	Intrinsic regulation of sport motivation was related to higher depressive symptoms.	Sheehan, Herring, and Campbell (2018)
		*N* = 236 healthy adolescents	Perceived Teacher Autonomy Support Questionnaire, General Basic Needs Satisfaction Scale.	Teacher autonomy support increased psychological needs satisfaction and intrinsic motivation for school engagement, which, in turn, was associated with decreased anxiety and depression scores.	Yu, Li, Wang, and Zhang (2016)
		*N* = 115 healthy children	Perception of Success, Enjoyment of the Practice of Sports, Achievement Motivation in Physical Education.	In 11-12-year-old children, skill mastery ‘intrinsic’ motivation training increased task enjoyment, perceived ability and effort, as well as baseline anxiety.	Cecchini et al. (2001)
Schizophrenia spectrum disorders	‘Negative symptoms' in schizophrenia, schizoaffective disorder, and other psychotic illnesses span a range of behaviors again underscored by a lack of self-generated initiation, not limited to alogia, avolition, social withdrawal and affective blunting.	*n* = 66 SCZ or SZA; *n* = 44 controls	Motivational Trait Questionnaire: 3 components of intrinsic motivation (personal mastery, competitive excellence, motivation related to anxiety).	In control subjects only, IM was related to cognitive performance. Both groups showed positive relationships between intrinsic motivation and approach and avoidance behaviors.	Barch, Yodkovik, Sypher-Locke, and Hanewinkel (2008)
		*N* = 120 SCZ	Quality of Life Scale: Sum of 3 items, purpose, motivation, and curiosity.	In patients who were at the start of outpatient psychosocial rehabilitation programs, IM mediated the relationship between neurocognition and psychosocial functioning.	Nakagami, Xie, Hoe, and Brekke (2008)
		*N* = 57 SCZ or SZA	Intrinsic Motivation Inventory.	Intrinsically motivating instructional techniques during difficult task learning increased intrinsic motivation for the task, self-efficacy and achievement.	Choi and Medalia (2010)
		*N* = 130 SCZ or SZA	Quality of Life Scale: Sum of 3 items, purpose, motivation, and curiosity.	In patients from 4 community-based, psychosocial rehabilitation programs in Los Angeles, USA, IM was dynamic over time. Baseline IM predicted improvements in neurocognition, and change in IM was associated with change in psychosocial functioning.	Nakagami, Hoe, and Brekke (2010)
		*N* = 18 SCZ; *n* = 17 healthy controls	Enjoyable stop watch timing task where subjects stop a watch at an exact time. In this task, the watch starts automatically and must be stopped with a single button press within 50 ms of the 5s time point. The total number of successful trials is continuously displayed. A control task is passive watch viewing with a single button press when the watch stops.	Participants with SCZ showed lower IM for the task. Lateral prefrontal cortex activity during the cue period was associated with higher IM.	Takeda et al. (2017)
		*N* = 75 SCZ	Quality of Life Scale: Sum of 3 items, purpose, motivation, and curiosity.	High IM related to greater metacognitive mastery in a sample of patients with chronic illness.	Vohs and Lysaker (2014)
		*N* = 32 SCZ in functional remission	Intrinsic Motivation Inventory for Schizophrenia Research.	IM was associated with metacognition and subjects with greater intrinsic motivation and metacognition improved.	Tas, Brown, Esen-Danaci, Lysaker, and Brüne (2012)
		*N* = 58 SCZ spectrum disorders	Quality of Life Scale: Sum of 3 items, purpose, motivation, and curiosity.	IM was linked to extraversion, neuroticism and negative symptoms in this all-male cohort.	Vohs, Lysaker, and Nabors (2013)
		*N* = 12 SCZ	Intrinsic Motivation Inventory.	Among patients in outpatient treatment, IM for a cognitive task was associated with performance.	Fervaha, Agid, Foussias, and Remington (2014)
		*N* = 166 SCZ spectrum disorders	Quality of Life Scale.	All participants attended psychosocial rehabilitation programs in a diverse urban community. IM fully mediated the relationship between functioning and negative, disorganized, and global symptoms, and partially mediated the relationship between positive symptoms and functioning.	Yamada, Lee, Dinh, Barrio, and Brekke (2010)
		*N* = 49 SCZ or SZA	Intrinsic Motivation Inventory for Schizophrenia Research.	Perceived program value was the only predictor of attendance and cognitive improvement increased with improvements in program interest. Motivational changes over time were variable between subjects.	Bryce et al. (2018)
		*N* = 125 psychotic disorder	Quality of Life Scale: Sum of 3 items, purpose, motivation, and curiosity.	IM mediated the relationship between poor metacognition and impaired functioning.	Luther et al. (2016)
		*N* = 40 FEP; *N* = 66 prolonged psychosis	Quality of Life Scale: Sum of 3 items, purpose, motivation, and curiosity; PANSS.	FEP patients had higher IM and lower amotivation levels than the prolonged psychosis group. IM was associated with lower amotivation in both groups.	Luther, Lysaker, Firmin, Breier, and Vohs (2015)
		*N* = 535 SCZ with comorbid SUDs	Quality of Life Scale: Sum of 3 items, purpose, motivation, and curiosity.	The IM measure was reliable for this cohort. IM was negatively associated with alcohol and drug use severity, and changes in IM over time predicted alcohol/drug use severity.	Bahorik, Eack, Cochran, Greeno, and Cornelius (2015)
		*N* = 858 SCZ; *N* = 576 SCZ with comorbid SUDs	Heinrichs-Carpenter Quality of Life Scale	IM was negatively related to the likelihood of any alcohol or substance use at baseline. Reduced IM was associated with greater likelihood of alcohol or substance use at 6-month follow-up, whereas greater IM was protective against drug use.	Bahorik, Greeno, Cochran, Cornelius, and Eack (2017)
		*N* = 71 SCZ spectrum disorders	Quality of Life Scale: Sum of 3 items, purpose, motivation, and curiosity; Intrinsic Motivation Inventory.	The two IM measures were not significantly correlated among patients in an outpatient rehabilitation program. Only the QLS IM score was associated with rehabilitation outcomes.	Choi, Choi, Felice Reddy, and Fiszdon (2014)
Parkinson's disease	Apathy- In Parkinson's disease (PD), apathy describes reduced interest and execution of goal-directed activities, unrelated to depressive emotional states or cognitive impairment. There is an absence of spontaneous auto-activation, or self-generated behavior. three subtypes of disrupted processing: ‘cognitive’, ‘emotional-affective’, and ‘auto-activation’.	*N* = 27 PD; *N* = 27 healthy controls	Curiosity for resolving uncertainty, despite negative outcomes, via choice to view or skip negative images.	The PD group viewed the images less frequently under the certain and uncertain conditions. The amount of pictures viewed was positively associated with the distribution of dopamine transporters in the striatum.	Shigemune et al. (2021)
		*N* = 28 PD	Participants stood on a stabilometer and aimed to maintain a horizontal platform position during each 30s trial, with the self-control group having autonomy to choose to use a balance pole while the yoked group used the balance pole on a set schedule.	The self-control group were more accurate and more motivated to learn the task compared to the yoked group.	Chiviacowsky, Wulf, Lewthwaite, and Campos (2012)
		*N* = 28 PD	Intrinsic Motivation Inventory.	In PD patients at general psychiatric outpatient clinics in Nanjing, those assigned to core stability training showed (1) higher IM compared to the home exercise group, and (2) increased interest and pleasure, perceived merit, effort and general motivation at the 8-week follow-up.	Sun and Chen (2017)
		*N* = 57 PD	Regulatory Mode Questionnaire.	Patients showed reduced assessment motivation only.	Foerde, Braun, Higgins, and Shohamy (2014)
SUD, AUD, and gambling disorder	One symptom of SUDs and AUD relates to individuals forgoing important work-related, social or recreational activities due to their substance use. Among others, this symptom relates to reduced goal-directed behaviors, which may indicate impaired IM.	*N* = 454 SUD	Circumstances, Motivation, Readiness, and Suitability instrument, Norwegian version.	In patients from 5 inpatient SUD centers in Norway, higher IM for changing substance use was associated with lower dropout risk.	Andersson, Steinsbekk, Walderhaug, Otterholt, and Nordfjærn (2018)
		*N* = 15 SUD adolescents; *N* = 15 caretakers	Interview about treatment experience coded for *4* dyadic categories: *intrinsic motivation*; *extrinsic motivation*; both or *mixed*/*transitional*; and disagreement/conflicting.	Adolescent patients with higher IM were more engaged in treatment.	Cornelius, Earnshaw, Menino, Bogart, and Levy (2017)
		*N* = 611 SUD	Reasons for Quitting Questionnaire adapted for use with substance users other than tobacco smokers.	Intrinsic self-concept issues were related to abstinence. IM was higher than IM in this sample of treatment-seeking individuals with poly-substance use disorders	Downey, Rosengren, and Donovan (2001)
		*N* = 252 undergraduate students	Gambling Motives Scale & General Causality Orientation Scale	In an at-risk sample, greater autonomy was associated with lower problematic gambling, in part, due to a lower tendency of chasing losses.	Rodriguez, Neighbors, Rinker, and Tackett (2014)
		*N* = 887 regular gamblers	Global Motivation Scale & Basic Psychological Need Satisfaction and Frustration Scale	Greater IM was weakly associated with increased problematic gambling.	Mills, Li Anthony, and Nower (2020)
		*N* = 94 undergraduate students	Intrinsic–Extrinsic Aspirations Scale.	IM and sense of control were positively associated with adaptive motivation and negatively with alcohol intake.	Shamloo and Cox (2010)
		*N* = 1137 smokers	Reasons for Quitting scale.	In this population-based sample, higher IM relative to EM was associated with greater readiness to quit and successful smoking cessation at 1-year follow-up.	Curry, Grothaus, and McBride (1997)
		*N* = 1961 adolescents	Ratings of emotional engagement.	In a diverse adolescent sample, positive time attitudes were indirectly associated with less marijuana use via IM, engagement, and less alcohol use. The indirect effect of positive time attitudes on engagement via IM was significant and substantial. Negative time attitudes and IM were indirectly associated with less marijuana use via behavioral engagement.	Froiland, Worrell, Olenchak, and Kowalski (2020)

*Note:* Cohort abbreviations: AUD, alcohol use disorder; FEP, first-episode psychosis; MDD, major depressive disorder; PD, Parkinson's disease; SCZ, schizophrenia; SUDs, substance use disorders; SZA, schizoaffective disorder. Evidence abbreviations: EM, extrinsic motivation; IM, intrinsic motivation.

In the Diagnostic and Statistical Model of Mental Disorders, 5th edition (DSM-*5*), anhedonia serves as one of two cardinal symptoms of depressive disorders, where it is defined as the ‘loss of interest or pleasure in all, or almost all, activities’, (American Psychiatric Association, 2013). The second cardinal symptom relates to persistent depressed mood. Approximately one-third of individuals with depression report clinically significant anhedonia (Pelizza & Ferrari, 2009), and these individuals are at-risk for poorer treatment outcomes, including nonresponse, relapse, and increased suicidality, relative to their non-anhedonic peers (Morris, Bylsma, & Rottenberg, 2009; Nierenberg et al., 1999).

Anhedonia remains an important clinical target that, by definition, implicates perturbations in intrinsically-motivated behavior, yet most empirical studies of anhedonia and motivation have investigated their relationship using extrinsic reinforcers. Findings broadly support theories of reward dysfunction in depression (reviewed by Sescousse, Caldú, Segura, and Dreher, 2013; Roiser & Husain, 2018; Borsini, Wallis, Zunszain, Pariante, and Kempton, 2020), where anhedonia has been associated with a reduced bias toward a monetary reward in individuals with depression (Liu et al., 2011) and their first-degree relatives (Liu et al., 2016). Children who are at-risk for depression show reduced VS and anterior insula responses to monetary gains, implicating blunted reward sensitivity as an antecedent to anhedonia (Luking, Pagliaccio, Luby, & Barch, 2016). Moreover, vmPFC responses during unexpected reward receipt may indirectly relate to anhedonia in depressed patients by modulating task motivation (Segarra et al., 2016). Interestingly, reward sensitivity disturbances in depression might not extend to aberrant reward learning (Huys, Pizzagalli, Bogdan, & Dayan, 2013) where adults with moderate depression show intact VS RPE-signaling during probabilistic learning (Rutledge et al., 2017). Nevertheless, there have been suggestions that perturbations in domains more related to intrinsic motivation, such as model-based future planning or effort initiation and invigoration, may be key in underlying anhedonia (Berwian et al., 2020; Cooper, Arulpragasam, & Treadway, 2018; Rutledge et al., 2017). Finally, affect can also alter both the valence and evaluation of an activity, which can, in turn, modulate the likelihood of selecting a more inherently interesting task (Isen & Reeve, 2006). Anhedonic individuals have more pessimistic likelihood estimates and reduced positive affective forecasts relative to controls while also demonstrating greater reliance on negative emotion during future-oriented cognition (Marroquín & Nolen-Hoeksema, 2015).

While few studies have implemented objective measures of intrinsic motivation in studying anhedonia, recent work links this symptom with difficulties with representations of future states during early stages of motivated behavior (Moutoussis et al., 2018). Since intrinsic motivation is driven more by proactive factors as opposed to the more reactive domain of extrinsic motivation, parsing future-oriented decision-making might provide novel insights not only into mechanisms of intrinsic motivation but also anhedonia. When considering the pre-decisional deliberation phase of motivated action (Fig. 1), the representation of a future state may be critical for distinguishing intrinsic *v.* extrinsic motivation. For example, disrupted representations of intrinsic reinforcers (e.g. autonomy, achievement, task enjoyment, novelty seeking), energy expenditure (Treadway, Cooper, & Miller, 2019; Winch, Moberly, & Dickson, 2014), or fatigue (Müller, Klein-Flügge, Manohar, Husain, & Apps, 2021) might disrupt choice deliberation and interrupt ensuing stages of motivation. This could critically determine the capacity for self-generated, intrinsically-motivated actions (Husain & Roiser, 2018). However, relatively few studies have examined this distinction. One study developed a cognitive task that aimed to capture separate measures of self-generated (*intrinsic*) *v.* externally generated (*extrinsic*) motivation during the option-generation phase (Morris et al., 2020). This distinction linked self-generated option generation (intrinsic motivation) to anhedonia symptoms in healthy adults (Morris et al., 2020). However, this task still relies on extrinsic rewards, and there is a need for improved tasks that index both behavioral and neural correlates of intrinsic drivers of motivated behavior.

## Summary and future directions

In this review, we summarize how intrinsic motivation has been conceptualized, measured, and related to neural function to elucidate its role in psychopathology. In contrast to extrinsic motivation, which has been rapidly incorporated into prominent cognitive, computational, and neurobiological models of human behavior, knowledge of intrinsic motivation remains limited due to evolving conceptualizations, imprecise measurement, and incomplete characterization of its biological correlates. We identify three potential areas of interest for future research.

First, additional objective measures of intrinsically motivation should be developed. This remains challenging experimentally since even the closest approximations of intrinsic motivation (Murayama et al., 2010; Rutledge et al., 2017) define the construct relative to extrinsic motivation, and other paradigms (e.g. exploration/exploitation tasks) rely on the presence of extrinsic reinforcers. Rather than defining motivated behavior as intrinsic or extrinsic, a more tractable approach might be to consider separate drivers of behavior that can be intrinsic or extrinsic. Future paradigms could index intrinsic motivation by characterizing the effects of intrinsic *v.* extrinsic reinforcers on motivation for an activity that is enjoyable. Such a design would enable more complex modeling of the effects of distinct reinforcers, and interactions between them, on motivated behavior, which would resolve inconsistencies surrounding the impact of extrinsic reinforcers on intrinsic motivation. For example, monetary incentives might reduce motivation only when a perceived agency is low, or when task enjoyment is high. These interactions might explain paradoxical observations like the undermining effect.

Second, computational models are needed to characterize intrinsic motivation. Computational models of motivation have been successfully implemented in studies of extrinsic motivation, yet few are appropriate for intrinsic motivation due to a focus on action-outcome associations. However, if the intrinsic reward were operationalized as a measurable outcome (e.g. completion of an enjoyable task), reinforcement-learning models could estimate how intrinsic reward value is represented. Advancements in the computational area could significantly improve understanding of the latent processes underlying (ab)normal decision-making, thereby identifying novel therapeutic targets.

Third, although evidence supports the bifurcation of intrinsic and extrinsic motivation at the psychological level, findings at the neural level are more equivocal. Given the overarching role of the mesolimbic dopamine system in learning, reward value estimation, and exploratory behavior, it is perhaps unsurprising that current evidence supports largely overlapping neural circuits for intrinsically and extrinsically motivated behavior. One potential avenue involves targeted pharmacological manipulations or neuromodulation of cortico-limbic circuits to determine if intrinsically and extrinsically motivated behaviors can be systematically modulated in humans. By elucidating the neural circuits of distinct motivational processes and their associations with specific symptom profiles, this approach would improve targeted interventions for highly heterogenous and debilitating disorders like depression.
